# Development, regeneration, and physiological expansion of functional β-cells: Cellular sources and regulators

**DOI:** 10.3389/fcell.2024.1424278

**Published:** 2024-07-09

**Authors:** М. B. Chernysheva, Е. S. Ruchko, М. V. Karimova, Е. A. Vorotelyak, А. V. Vasiliev

**Affiliations:** ^1^ Cell Biology Laboratory, Koltzov Institute of Developmental Biology, Moscow, Russia; ^2^ Department of Biology and Biotechnologies Charles Darwin, The Sapienza University of Rome, Rome, Italy

**Keywords:** β-cell, differentiation, heterogeneity, regeneration, proliferation, diabetes, pancreas

## Abstract

Pancreatic regeneration is a complex process observed in both normal and pathological conditions. The aim of this review is to provide a comprehensive understanding of the emergence of a functionally active population of insulin-secreting β-cells in the adult pancreas. The renewal of β-cells is governed by a multifaceted interaction between cellular sources of genetic and epigenetic factors. Understanding the development and heterogeneity of β-cell populations is crucial for functional β-cell regeneration. The functional mass of pancreatic β-cells increases in situations such as pregnancy and obesity. However, the specific markers of mature β-cell populations and postnatal pancreatic progenitors capable of increasing self-reproduction in these conditions remain to be elucidated. The capacity to regenerate the β-cell population through various pathways, including the proliferation of pre-existing β-cells, β-cell neogenesis, differentiation of β-cells from a population of progenitor cells, and transdifferentiation of non-β-cells into β-cells, reveals crucial molecular mechanisms for identifying cellular sources and inducers of functional cell renewal. This provides an opportunity to identify specific cellular sources and mechanisms of regeneration, which could have clinical applications in treating various pathologies, including *in vitro* cell-based technologies, and deepen our understanding of regeneration in different physiological conditions.

## 1 Introduction

There are several pathologies characterized by a decrease in the cell mass of insulin-producing β-cells up to an absolute deficiency, namely T1DM (type I diabetes mellitus), certain forms of MODY (maturity-onset diabetes of the young), or surgical interventions. Alternatively, insulin resistance in peripheral tissues such as the liver, adipose and muscle tissues and consequent β-cell dysfunction result in T2DM (type II diabetes mellitus). Thus, over the years, studies focusing on discovering mediators of β-cell proliferation, survival, and functional mass preservation have remained relevant. These studies investigate β-cell population formation during ontogenesis, as well as responses to changes in physiological states such as pregnancy, obesity, or partial pancreatectomy (PPx).

The main mechanisms for pancreatic β-cell population generation include the proliferation of existing β-cells. In particular, β-cell replication is reduced in adults to an almost undetectable level (Dor et al., 2004; Perl et al., 2010; Xiao et al., 2013; Zhao et al., 2021). Other significant mechanisms include neogenesis from undifferentiated endocrine progenitor cells and transdifferentiation from ductal cells, α-cells, and other cell types (Inada et al., 2008; Nakamura et al., 2011; Furuyama et al., 2019; Gribben et al., 2021). A comprehensive understanding of these processes requires knowledge of the fundamental stages of pancreatic development, particularly the emergence of endocrine β-cells from the endoderm.

In recent years, there has been an increase in the number of studies examining the pancreatic β-cell heterogeneity phenomenon. Some cell populations are more sensitive to regeneration-inducing factors and stress agents. Additionally, certain cell populations can survive under stressful conditions, making them a promising source for regenerating the population of healthy, functionally active cells. We suggest that the phenomenon of β-cell heterogeneity is closely related to the diversity of potential regeneration scenarios. Also, understanding the complete variability of the cellular composition of native islets of Langerhans may be useful in studies of the differentiation of stem cells into insulin-producing cells *in vitro*. This is directly involved in solving the problem of lacking many functional characteristics of native β-cells, which was particularly common in early studies of the generation of insulin-producing cells.

There are various debates regarding the mechanisms of β-cell regeneration in the pancreas: neogenesis, proliferation, or transdifferentiation (Zhou and Melton, 2018; Spears et al., 2021). While the main inducing factors and potential cellular sources involved in the regeneration of the cell population producing insulin are recognized, it nevertheless remains an open question which of these mechanisms or their combinations play a pivotal role in specific circumstances.

## 2 The pancreas and the main stages of its development

The pancreas is an organ in the abdominal cavity composed of two functionally and morphologically distinct compartments originating from the endoderm and connected to the duodenum (Avolio et al., 2013; Dolenšek et al., 2015). The exocrine compartment is composed of acinar cells, which produce digestive enzymes, and a duct system. The endocrine compartment constitutes 1%–4% of the pancreatic tissue and is organized into highly vascularized and innervated clusters of cells called pancreatic islets of Langerhans, which include five different subtypes of hormone-secreting cells: α-, β-, δ-, ε-, and PP-cells, secreting glucagon, insulin, somatostatin, ghrelin, and PP (pancreatic polypeptide), respectively (Avolio et al., 2013). The high degree of islet vascularization enables the regulation of hormone release and the maintenance of the delicate control of glucose homeostasis in the body.

The ways in which the pancreas develops in humans and in rodents are similar but not identical (Kim et al., 2009; Jennings et al., 2013; Larsen and Grapin-Botton, 2017). Importantly, protocols for differentiation of pluripotent stem cells (PSCs) into insulin-secreting β-like cells are based on emulation of normal pancreatic development. Such key stages of embryonic development as the formation of the definitive endoderm, primitive intestinal tube, posterior foregut, and then pancreatic precursors and finally hormone-expressing endocrine cells are all remodeled by sequential treatment of PSCs with different combinations of growth and signaling molecules added to culture medium (Veres et al., 2019; Balboa et al., 2022). Nevertheless, the largest amount of data has been obtained in studies of pancreatic cell differentiation in mice. The majority of pancreatic cell types (acinocytes, ductal cells, and endocrine cells) differentiate during embryonic development from multipotent progenitor cells that coexpress the markers: pancreatic and duodenal homeobox 1 (Pdx1), pancreas associated transcription factor 1a (*Ptf*1*a*), NK6 homeobox 1 (*Nkx6*.1), and SRY-Box transcription factor 9 (*Sox9*) (Ramond et al., 2018; Aigha and Abdelalim, 2020). Cells that no longer express Sox9 become endocrine progenitor cells, they detach from the epithelium, and are found near or within primitive ducts (Meier et al., 2010), and are characterized by transient expression of neurogenin-3 (*Neurog*3) (Jennings et al., 2013). However, it is worth noting that *SOX*9 is important for *NEUROG*3 expression, possibly initiating transient expression of *NEUROG*3. It is known that *SOX*9 is commonly co-localized with *FOXA*2, *NEUROG3*, and *NEUROG*3-related transcription factors. There is also a suggestion that *NEUROG*3^+^ cells are a subpopulation of *SOX*9^+^ bipotent progenitors. *SOX*9 is also most frequently expressed in *PDX*1^+^ cells and least in mature endocrine cells. Knockdown of *SOX9* leads to a significant decrease in islet epithelial cell proliferation, decreased numbers of *NEUROG*3^+^ and *INS* ^+^ cells (McDonald et al., 2012).

Preserved postnatal endocrine progenitors may be a potential source of new β-cells in the adult organism. Endocrine *Neurog*3^+^ progenitor cells differentiate into all five types of adult pancreatic endocrine cells (α-, β-, δ-, ε-, and PP-cells) during embryogenesis (Gradwohl et al., 2000; Heller et al., 2005). Inhibition of *Neurog*3 in the pancreas on day 11 of embryonic development (E11) in mice results in a significant reduction in endocrine differentiation (Prasadan et al., 2010). *Neurog*3 gene knockout mice have no islet endocrine cells, and in humans, known *NEUROG*3 mutations contribute to diabetes development to varying degrees (McGrath et al., 2015; Zhang et al., 2019). In addition to *NEUROG*3, endocrine progenitor cells in both mice and humans continue to express *PDX*1 and *NKX*6.1, but they do not yet express hormone genes (Zhu et al., 2016; Yu and Xu, 2020). In mice, the differentiation process of endocrine cells is divided into two waves according to Ngn3 expression. The first period is associated with the generation of early α-cells (E9.5–E12.5), while the second (from E12.5 to birth) is associated with the formation of the remaining islet cell types (Yu et al., 2019). Only one wave of differentiation has been described in human development (Villasenor et al., 2008; Jennings et al., 2013; Salisbury et al., 2014). *NEUROG*3 expression peaks around the end of the first trimester, which is associated with the appearance of fetal β-cells characterized by the expression of NK2 homeobox 2 *NKX*2.2, *NKX*6.1, *PDX1*, *ISL LIM homeobox* 1 (ISL1), human insulin (*INS*) and forkhead box A2 (*FOXA*2) (which is expressed during prior developmental stages starting from epithelial cells of the endoderm) (Jennings et al., 2013; Moin and Butler, 2019), whereas after 35 weeks of development, NEUROG3 expression is undetectable (Salisbury et al., 2014). β-cells have been detected in the human embryo from week nine of gestation, while glucagon expression has been detected from week 8 onwards (Meier et al., 2010). By the 10th week of development, clusters of β-cells differ in their vascularization, and during weeks 12–13, all cell types are already distinguished in the islets. After the initiation of insulin and glucagon expression, the expression of Maf family transcription factors, MAF bZIP transcription factor A (*Mafa*) and MAF bZIP transcription factor B (*Mafb*), is detected in pancreatic tissue. These factors are crucial for the development and maturation of endocrine cells (Vanhoose et al., 2008; Yu and Xu, 2020). In the murine embryonic pancreas, a significant number of cells that produce insulin also express *Mafb*. These cells go through the *Mafb* ^+^ and *Mafa* ^+^ expression phases during the maturation of β-cells, before fully maturing into *Mafb* ^−^ and *Mafa* ^+^ β-cells. In humans, *MAFA* and *MAFB* expression patterns in β-cells differ from those in rodents. Overall, *MAFA* expression levels increase from the embryonic period to adulthood (Blodgett et al., 2015). The most distinctive feature in comparison to rodents is the expression of *MAFB* in mature human β-cells (Cyphert et al., 2019).

In most vertebrate species, β-cells form clusters with other hormone-secreting cells within pancreatic islets. The endocrine compartment of the pancreas contains 1–15 million pancreatic islets in humans and 1–5 thousand pancreatic islets in mice (Dolenšek et al., 2015). In newborn children, the islets have equal numbers of α-, β-, and δ-cells, but in the first 2 years of life, β-cells proliferate and become the most numerous, while the number of δ-cells decreases markedly (Gregg et al., 2012). The architecture of human and murine islets differs. In the human pancreas, β-cells appear to be mixed throughout the islet. In murine islets, β-cells are concentrated in the core of each islet and surrounded by peripheral endocrine cells, mostly α-cells. β-cells make up to 75% of all pancreatic islet cells (Dolenšek et al., 2015; Ionescu-Tirgoviste et al., 2015; Overi et al., 2022). The remaining cells are located in the periphery, forming a mantle where α-cells are predominant (Kim et al., 2009). Human islets have a relatively small proportion of β-cells, compared with other species. Moreover, as islet size increases, the proportion of β-cells in human islets decreases. In humans and mice, different cell types are unevenly distributed in different parts of the pancreas: the highest concentration of PP cells is in the head of the pancreas (known as the PP-rich lobe), where 90% of all PP-cells are located, while the remaining areas (neck, body, and tail) are characterized by a higher proportion of α- and β-cells. There is a close correlation between body mass and pancreatic cell mass (Brereton et al., 2015). The total mass of β-cells also increases proportionally (Kim et al., 2009). Pancreatic islets vary considerably in size up to 500 μm, but a typical islet is about 50–200 μm in diameter (Dolenšek et al., 2015; Ionescu-Tirgoviste et al., 2015). It should be noted that pancreatic volume and size are reduced in T1DM patients, and this is affected by the duration and age of disease onset (Ragimov et al., 2022).

To summarize, pancreatic islets form during embryonic development by delaminating from the pancreatic duct. There are some differences in endocrine cell formation and pancreatic islet architecture between mice and humans (Kim et al., 2009; Meier et al., 2010). The development of the pancreas and the origin of endocrine β-cells from the endoderm are coordinated by a complex interplay of signaling pathways and transcription factors. This is described in detail in a review by Oliver-Krasinski and Stoffers (Oliver-Krasinski and Stoffers, 2008).

## 3 Functional maturity and heterogeneity of β-cells

**FIGURE 1:**
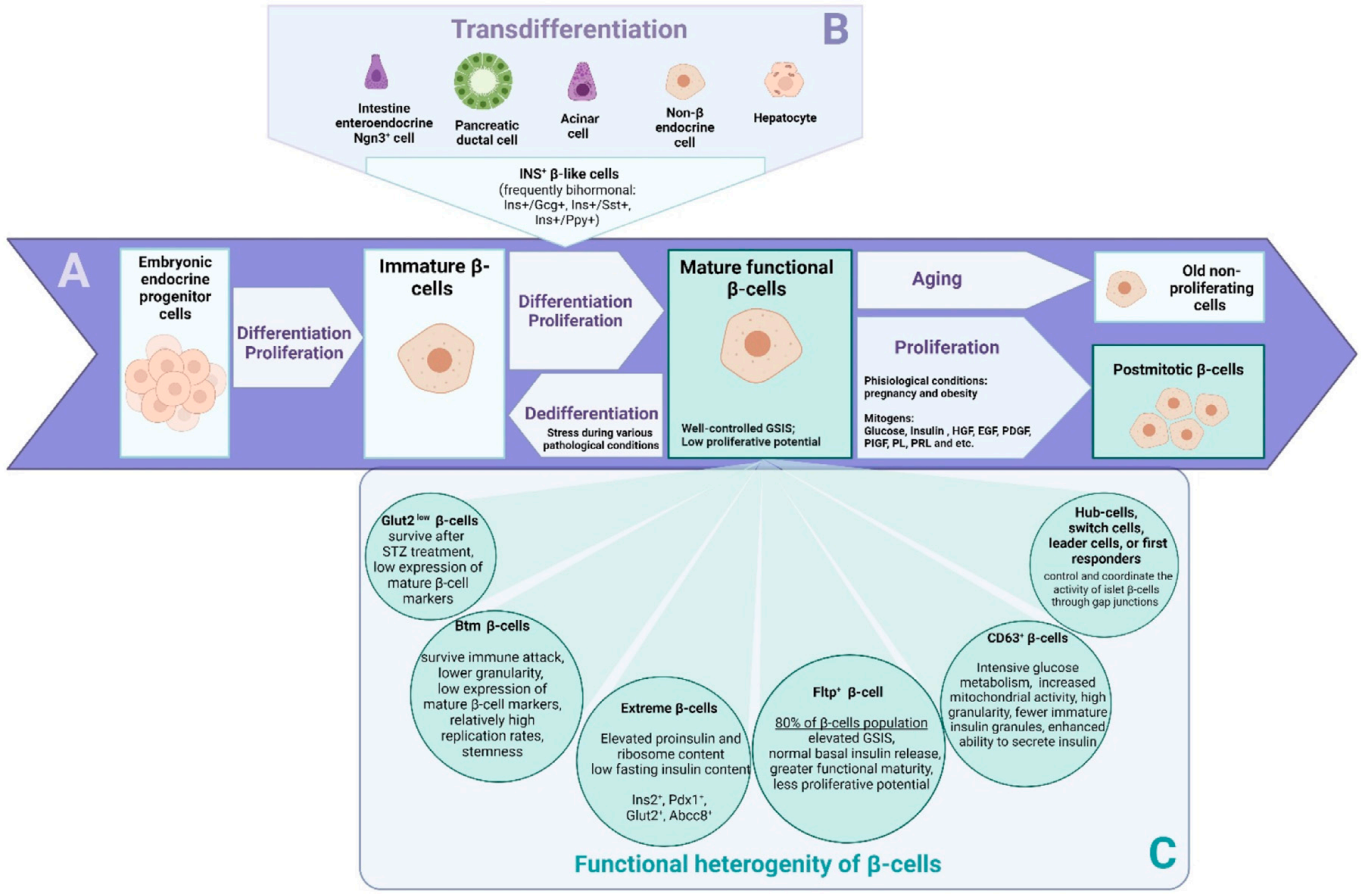
The β cells that exist in the body are a primary source for the generation of a population of functionally active β cells. Proliferation **(A)** is most active during neonatal stages, while in adulthood, the rate of β-cell proliferation is low and decreases with age. There are two additional scenarios for the generation of new β-cells in the adult organism: neogenesis of β-cells from undifferentiated endocrine progenitor cells, and transdifferentiation **(B)** from other cell types to β-cells. The mechanisms and cellular sources of β-cell regeneration in the pancreas are controversial. Immature β-cells or some populations of mature, functionally low-active cells may potentially serve as a source for renewing the mass of functionally active β-cells. In recent years, it has become possible to identify distinct subpopulations **(C)** of pancreatic cells with different transcriptomic profiles and key marker genes. The heterogeneity of β-cells allows the observation of different scenarios.

There are several perspectives for understanding the phenomenon of β-cell population heterogeneity. One set of data highlights the heterogeneity of β-cells due to their varying degrees of differentiation and maturation in the adult organism (Figure 1A). Another set of studies aims to establish the relationship between gene expression and its phenotypic manifestation in β-cell subpopulations (Figure 1C). The third set of studies examines β-cells as multicellular networks of interconnected cells. Recently, it has become possible to identify different subpopulations of pancreatic cells that differ both in their transcriptomic profile and in key marker genes (Blum et al., 2012; Tremmel et al., 2023). This allows researchers to better understand the genesis of functionally active populations of β-cells in adult pancreatic islets and possible cell sources for their repair. Transcriptomic studies have also shed light on the heterogeneity of the pancreatic β-cell population (Baron et al., 2016).

It is known that in the postnatal period, immature β-cells transform into fully mature and functionally active cells capable of responding to and controlling blood glucose levels through glucose-sensitive insulin secretion (GSIS). Maturation of β-cells in mice correlates with an increase in the expression levels of genes such as mouse insulin (*Ins*2), *Mafa*, solute carrier family two member 2 (*SLC*2*A*2, formerly known as *Glut*2), *Pdx*1, *Nkx*6.1, neuronal differentiation 1 (*Neurod*1), and urocortin 3 (*Ucn*3). Notably, adult terminally differentiated β-cells are characterized by a canonical expression profile of major genes such as *Ins*, *Mafa*, *SLC2A*2, *Pdx*1, *Nkx*6.1, *Neurod*1, *Nkx*2.2, and cyclin dependent kinase inhibitor 2A (*Cdkn*2*a*, formerly known as p16INK4a) (Avrahami et al., 2015; Xin et al., 2016). In the adult organism, the β-cell population is renewed very slowly, so at any given time, cells of different ages are present in the tissue. Immature β-cells have a high proliferative potential (Figure 1A). They do not respond well to glucose stimulation, but they do secrete some insulin even when basal blood glucose levels are low. Immature β-cells are characterized by the expression of hexokinase 1 (*Hk*1), paired box 6 (*Pax*6), and *Mafb*. Through transcriptome analysis, features of immature, proliferative β-cells such as a high expression of metabolic amino acid genes, as well as of mitochondrial and serum response factor (*Srf*), Jun proto-oncogene (*Jun*), Fos proto-oncogene (*Fos*) transcription factors, have been identified (Zeng et al., 2017). Mature cells rarely proliferate and intensively secrete insulin in response to external stimuli such as glucose (Andrali et al., 2008), several amino acids such as leucine, isoleucine, valine, lysine, threonine (Nilsson et al., 2007), and incretins (Marrano et al., 2021) (Figure 1A). The main characteristic of mature and functionally active β-cells is well-controlled GSIS. Old cells stop proliferating and may become functionally inactive, meaning they are primarily unable to secrete insulin (Bonner-Weir and Aguayo-Mazzucato, 2016). In view of the above, immature beta cells are the focus of interest for the promotion of proliferation and expansion of the population of cells that secrete insulin (Figure 1A).

At the gene level, the functional maturation of rodent and human β-cells, in addition to the accompanying rise of the glucose threshold for insulin secretion, is associated with increased expression levels of a number of genes (Blum et al., 2012). UCN3 has long been postulated as a marker of mature β-cells (van der Meulen et al., 2012). However, recent studies suggest that UCN3 expression does not correlate with the functional maturation of human β-cells (Tremmel et al., 2023). UCN3 is expressed in human fetal islets long before functional maturation is reached. Islets derived from human embryonic stem cells express significant levels of UCN3, but by the end of differentiation (day 28), the generated pancreatic islets show low levels of GSIS. Neuropeptide Y (NPY) is a marker of functionally immature β-cells. The functional maturation of β-cells is characterized by decreased replication intensity and Npy expression (Meier et al., 2008; Rodnoi et al., 2017). Reduced Npy expression levels in the islets of newborn mice correlate with decreased proliferation and enhanced GSIS (Rodnoi et al., 2017). According to Tremmel and colleagues, the expression of the chromogranin B (*CHGB*), glucose-6-phosphatase catalytic subunit 2 (*G*6*PC*2), shisa like 2B (*SHISAL*2*B*), *SLC*2*A*1, islet amyloid polypeptide (*IAPP*), and ectonucleoside triphosphate diphosphohydrolase 3 (*ENTPD*3) genes correlates with the onset of functional maturation of human β-cells, while the expression of endoplasmic reticulum oxidoreductase one beta (*ERO*1*LB*), histone deacetylase 9 (*HDAC*9), KLF transcription factor 9 (*KLF*9), and solute carrier family 30 member 10 (*ZNT*8) does not change between fetal and adult developmental stages (Tremmel et al., 2023). Also, it was shown that *ISL1*, a LIM-homeodomain transcription factor involved in the epigenetic control of embryonic development, transcriptionally and epigenetically controls α-cell differentiation and β-cell maturation (Bohuslavova et al., 2023). *ISL*1 controls the formation of the mature phenotype of β-cells. The authors also suggest that *ISL*1 regulates *PDX*1 expression during pancreatic β-cell maturation (Bohuslavova et al., 2023). In the case of β-like cells differentiated from PSCs, it is important to note that immature cells are also present in the population, underscoring the importance of the transplantation step of differentiated cell clusters into animal models to promote cell maturation *in vivo* as well as to perform screening and sequencing of grafts (Pellegrini et al., 2022).

One of the common methods of enriching the population of differentiated β-like cells is cell sorting, which allows to exclude undesirable and immature cell types by analyzing cell surface markers associated with positive or negative β-cell features. Many studies looking for markers of β-cell subpopulations and their correlation with β-cell maturation and functional characteristics are based on the phenotypic expression of specific genes. A recent study discovered the presence of four clusters of β-cells by scRNA-sequencing (scRNA-seq) and their functional maturity marker, *CD*63 (Rubio-Navarro et al., 2023). Functionally, β-cells with high *CD*63 expression exhibit intensive glucose metabolism, increased mitochondrial activity, high granularity, fewer immature insulin granules, and an enhanced ability to secrete insulin. Another group of researchers focused on *CD*81 as a marker. High *CD*81 expression is characteristic of immature β-cells and gradually decreases until it completely disappears in β-cells during postnatal maturation. The reverse process–an increase in *CD*81 expression - was observed in cases of diabetes or stress (Salinno et al., 2021).

The endocrine cells of human pancreatic islets can be divided into four subpopulations (Dorrell et al., 2016). The authors reported that they can be distinguished by differential expression of two surface antigens: ST8 alpha-N-acetyl-neuraminide alpha-2,8-sialyltransferase 1 (*ST*8*SIA*1) and *CD*9. These subpopulations have different gene expression profiles as well as distinct basal insulin secretion and GSIS levels. *ST*8*SIA* ^+^ pancreatic cells are characterized by higher levels of GSIS, while *ST*8*SIA* ^−^ pancreatic cells have increased basal insulin secretion. The distribution of β-cell populations is constant in healthy individuals, but in patients with type 2 diabetes, there is a significant decrease in the number of the more functional *CD*9^−^ *ST*8*SIA*1^−^ β-cells (25%), compared with healthy individuals (∼50%). Conversely, there is a significant increase in the number of less functional *CD*9^+^ *ST*8*SIA*1^+^ β-cells in patients with T2DM (25%), compared with healthy individuals (∼10%). In contrast to the results of Bader and colleagues, there is no data on the origin of these subpopulations. Therefore, it remains to be determined whether conversion from one population to another occurs (Bader et al., 2016; Dorrell et al., 2016).

Cilia and flagella associated protein 126 (*Cfap*126, formerly known as Fltp), a Wnt/planar cell polarity effector, has recently been shown to distinguish young proliferating cells from mature β-cells with different molecular, physiological, and ultrastructural features. Genetic lineage tracking revealed that endocrine subpopulations from Fltp^−^ and Fltp^+^ lines respond differently to physiological and pathological changes. The Fltp^+^ population of mature β-cells is characterized by increased GSIS but normal basal insulin secretion, greater functional maturity, and lower proliferative potential, accounting for 80% of β-cells, while Fltp^−^ populations have greater proliferative capacity (Bader et al., 2016). During periods of metabolic stress, such as pregnancy, Fltp^−^ β-cells are able to proliferate and then change their expression profile to Fltp^+^ for better management of metabolic changes (Bader et al., 2016).

There is a hypothesis that β-cell populations differ in their resistance to different kinds of ablation. Thus, it is known that detectable insulin concentrations are still present in some people with long-standing diabetes, as autoimmune destruction of human β-cells in T1DM patients is not always complete. It is also known that inflammation in T1DM is not uniform throughout the islet, and some regions may be free of inflammation (Atkinson et al., 2011). β-cells store insulin in dense secretory granules, membrane-bound organelles filled with insulin. A subpopulation of cells with a lower granularity (Btm) has been identified as resistant during insulitis. During the progression of T1DM in non-obese diabetic mice (NOD mice), β-cells of the Btm subpopulation are characterized by a reduced insulin content. The Btm cell subpopulation demonstrates features of dedifferentiated β-cells, a lower granularity of insulin, a low expression of mature β-cell markers, and relatively high replication rates. As insulitis progresses, the frequency of Btm β-cells increases while the expression of genes associated with apoptosis and cell death decreases. This suggests that Btm β-cells are more resistant to autoimmune β-cell destruction during T1DM. The Btm population is undetectable in mice without insulitis (Rui et al., 2017). A population of “extreme β-cells” is identified in pancreatic islets in transgenic mouse models. The proportion of “extreme β-cells” increases in insulin-resistant animals (db/db mice). This cell population is characterized by high mRNA levels of *Ins*2, *Pdx*1, *Slc*2*a*2, and ATP-binding cassette subfamily C member 8 (*Abcc*8), as well as increased content of proinsulin and ribosomes, but low content of insulin in the fasting state. Moreover, the mRNAs are located in the apical part of the cells, separately from the insulin granules. Presumably, this β-cell population specializes in basal insulin secretion; rather than accumulating granules inside the cells, they preferentially secrete them when blood glucose levels are normal (Farack et al., 2019). Another group of researchers identified two β-cell populations by scRNAseq: a large population with a high expression of glucose transporter 2 (Glut2 ^high^, known now as *Slc2a2*) and a small population with a low Glut2 expression (Glut2^low^) (Feng et al., 2020). At the transcriptomic level, the population of Glut2^low^ β-cells was not characterized as immature. However, some of the genes associated with β-cell maturation and function were decreased in these cells. After a single injection of a high dose of streptozotocin (STZ), a diabetogenic chemical agent that enters β-cells via Glut2, causing DNA alkylation and activation of poly-ADP-ribosylation leading to a depletion of cellular NAD^+^ and ATP, most cells with a Glut2^high^ content died, but cells with a Glut2^low^ content survived. Surviving cells eventually transitioned to an undifferentiated state. However, no conversion of Glut2^low^ to Glut2^high^ β-cells was observed for up to 9 months after STZ exposure (Feng et al., 2020).

Analysis of the pancreatic cell transcriptome also shows that β-cells differ in the expression levels of the genes that are activated during endoplasmic reticulum (ER) stress and unfolded protein response (UPR): homocysteine-inducible ER protein with ubiquitin-like domain 1 (*HERPUD*1), heat shock protein family A (*Hsp*170) member 5 (*HSPA*5), and DNA damage-inducible transcript 3 (*DDIT3*) (Baron et al., 2016). ER stress and UPR are caused by an increase in insulin production. A link between UPR and the proliferation of murine and human β-cells has been shown. UPR affects not only ER function at the single cell level, but also cell proliferation throughout the pancreas (Sharma et al., 2015). In the case of studying populations of β-cells using scRNAseq-derived data, it is worth noting that despite their uniqueness and utility, different states of a single population of β-cells can be characterized using transcriptome analysis, but not with absolute certainty to identify distinct subpopulations of β-cells.

Early studies of β-cells assumed that they were homogeneous due to a lack of morphological differences. However, detailed studies have shown that β-cells in pancreatic islets of the adult pancreas are heterogeneous in size, granularity, transcriptional profile, and function (Katsuta et al., 2012). The ratios of β-cell populations also change during diabetes (Dorrell et al., 2016; Benninger and Kravets, 2022). Furthermore, β-cells are known to differ in their insulin release rates, glucose and glucokinase metabolism, insulin expression, membrane potential value and Ca^2+^ content, cAMP, and NAD(P)H signaling (Benninger and Kravets, 2022; Rubio-Navarro et al., 2023). Only 20% of β-cells secrete almost all the amount of insulin, which is most likely due to contacts between β-cells and their position in islets, in other words, the existence of spatial and temporal cell coordination (Wojtusciszyn et al., 2008; Almaça et al., 2015).

Pancreatic islets function as multicellular networks. It is widely accepted that a subpopulation of cells, known as hub cells, exhibits a high proportion of functional connections with other cells (Johnston et al., 2016; Salem et al., 2019; Šterk et al., 2023). Hub cells’ functional connections greatly enhance the communication capabilities of the intercellular network of pancreatic islets and promote efficient propagation of intercellular Ca^2+^ waves (Šterk et al., 2023). Hub cells control the activity of all islet β-cells through gap junctions and coordinate synchronous oscillations of intracellular calcium and insulin secretion with high glucose levels. Silencing the functions of these pacemaker cells should result in a loss of islet functional activity (Hogan and Peercy, 2021). Johnston and colleagues showed that only 1%–10% of β-cell hubs regulate GSIS (Johnston et al., 2016). This phenomenon is related to the fact that hub cells exhibit earlier Ca^2+^ oscillations and are able to identify Ca^2+^ dynamics among other cells within the whole islet that exhibit a slower frequency of Ca^2+^ oscillations. Research by Dwulet and colleagues suggested that a subpopulation of hub β-cells that exceeds 30% of all β-cells in the islet is required to maintain elevated levels of Ca^2+^ and insulin release (Dwulet et al., 2021). In addition, recent studies have identified other cell types that do not have as many connections as hub cells, yet they are the ones that trigger intercellular signaling: switch cells, leader β-cells, and first responder cells in both mouse and human islets (Johnston et al., 2016; Salem et al., 2019; Hogan and Peercy, 2021). Systematically silencing each cell in the network revealed switch cells that could deactivate the islet’s activity but were not highly functionally connected (Hogan and Peercy, 2021). Salem and colleagues discovered that Ca^2+^ spikes come from a specific group of β-cells, named “leader-cells”. They are the first to respond to a glucose stimulus (Salem et al., 2019). Šterk and colleagues have identified subpopulations of wave-initiator cells, often described as pacemaker cells. These cells were identified during the initial transient phase when they responded to stimulatory glucose levels. It is important to note that the wave-initiator cells should not be confused with the first responder cells, which trigger the repetitive intercellular waves during sustained activity (Šterk et al., 2023). Thus, not only the heterogeneity of β-cells but also the percentage of their subpopulations can influence the dynamics of insulin secretion.

It is of great interest how subpopulations such as Btm cells, “extreme β-cells”, *CD*63^low^, *SLC*2*A*2^low^, *CD8*1^high^ or *ST*8*SIA* - β-cells relate, and which of these markers and their combinations are most specific for immature, proliferation-capable, and insulitis-resistant cells (Figures 1A,C). The diversity of β-cell populations suggests that regeneration mechanisms may also significantly differ in various pathologies and scenarios of β-cell regeneration, as discussed in the following chapters of our review. Perhaps the accumulation of knowledge about the differences between β-cells in the context of their sensitivity to stress is a direct way to develop a strategy for diabetes therapy based on the maintenance or expansion of the population of immature β-cells and their further maturation for the regeneration of the population of insulin-secreting cells.

## 4 Regeneration of the pancreas

The pancreas has the ability to regenerate under normal and pathological conditions. The regeneration of β-cells can be roughly divided into three categories: 1) proliferation of existing β-cells; 2) β-cell neogenesis or β-cell differentiation from the progenitor cell population; and 3) transdifferentiation of non-β-cells into β-cells (Dor et al., 2004; Inada et al., 2008; Perl et al., 2010; Nakamura et al., 2011; Xiao et al., 2013; Gribben et al., 2021; Zhao et al., 2021). However, it remains unclear which mechanisms of β-cell population reconstitution are activated during pancreatic regeneration in humans. It is possible that different cellular sources participate in β-cell regeneration in response to damage of varying degrees of intensity and differing origins. Cooperative action cannot be ruled out.

Experimental animal models have been extensively used to study pancreatic β-cell regeneration in the context of therapies for T1DM and T2DM. Chemically induced diabetes rodent models are primarily used due to easily induced diabetes, a short induction period, and cost-effectiveness (Furman, 2021). Relevant data on such rodent models are summarized in reviews by Gvazava et al. (Gvazava et al., 2018; 2022). However, traditionally, models based on pancreatic duct ligation (PDL) and partial pancreatectomy were used to study β-cell regeneration, demonstrating the fundamental possibility of pancreatic regeneration. Tissue regeneration shares common features with embryonic development. It is assumed that embryonic programs are partially reactivated in the adult organism to build new tissue and regenerate islets. In a significant paper by Bonner-Weir and colleagues, the incorporation of bromodeoxyuridine (BrdU) into cells in the ducts after 90% PPx in Sprague-Dawley rats demonstrated a high level of proliferation (Bonner-Weir et al., 1993; Goldman and Poss, 2020). It has been reported that regeneration is first observed in the common pancreatic duct and then in the smaller ducts of the ductal tree. These smaller ducts differentiate into newly formed pancreatic islets. In a recent study, Overi and colleagues (Overi et al., 2022) observed that a significant regenerative process may occur in T2DM patients. They provided evidence that regenerating islets may be associated with the pancreatic duct glands cell niche. The authors discovered the presence of islet-like structures within the pancreatic duct gland compartment in the pancreatic ducts of T2DM patients. These structures contained insulin- and glucagon-positive cells. In mice treated with STZ, pancreatic duct gland proliferation was also observed. This pathway follows embryonic development: proliferation of ductal cells is followed by differentiation of the exocrine and endocrine compartments of the pancreas near primitive ducts.

### 4.1 Is β-cell neogenesis possible as a regeneration scenario?

The mechanisms involved in pancreatic regeneration are a highly debated topic. Several studies have refuted the possibility of neogenesis in pancreatic regeneration, suggesting that this mechanism is difficult to activate or is extremely rare (Lee et al., 2011; Gregg et al., 2012; Cavelti-Weder et al., 2013; Rankin et al., 2013; Liu et al., 2021). For example, Lee, Hao, and Levine did not detect neogenic islets after PPx (Lee et al., 2011). The authors used continuous injection of BrdU to study β-cell regeneration in ICR mice after repeated PPx to find out which mechanism of population recovery prevails in this model: neogenesis or replication of β-cells. The researchers did not confirm the neogenesis of islet cells. According to these data, islets in the regenerating areas developed from pre-existing islets rather than being neogenic, as they were negative for BrdU inclusion 1 week after PPx. After repeated PPx, the regions of regeneration were devoid of islets. β-cell replication was detected with a high frequency 2 weeks after PPx and was present with the same frequency in both regenerating and pre-existing pancreatic sections. *INS* ^*+*^ cells were present in the ducts in the regenerating pancreatic sections but were relatively rare and not highly proliferative. Cavelti-Weder and colleagues created a complex model in Lewis rats combining depletion of the β-cell population with streptozotocin and subsequent PDL (Cavelti-Weder et al., 2013). The resulting severe form of diabetes was corrected by transplantation of pancreatic islets under the renal capsule before PDL. The authors observed regeneration of the ducts and acini of the exocrine compartment of the pancreas. However, there was no recovery of pancreatic islets and β-cells. This was evidenced by a lack of increase in the proportion of islet cells and low insulin content. In another study, Liu and colleagues obtained data suggesting that under normal physiological conditions, including pregnancy, duct cells and even *Ins* ^+^ duct cells make little or no contribution to the population of functional β-cells (Liu et al., 2021). Early studies using genetic tracking of cell lines also showed that the generation of new β-cells in mice is associated with the proliferation of existing β-cells, and specific β-cell progenitor cells are not detected (Dor et al., 2004; Teta et al., 2007).

The main unresolved problem within the concept of neogenesis has been (and still is) the identification of duct-like progenitors. It is known that pancreatic β-cells develop from a population of endocrine progenitors that reside in the pancreatic duct epithelium and express the transcription factor *Ngn*3, which labels pancreatic endocrine progenitors and responds to Notch signaling (Valdez et al., 2016; Gribben et al., 2021). The reactivation of the embryonic endocrine cell differentiation program in adult pancreatic ducts is of great interest and relevance, as it could serve as a potential source of new β-cells.

Neogenesis is evidenced by a number of studies, where the reappearance of *Ngn*3^+^ progenitor cells in the duct epithelium and the presence of small clusters of endocrine cells near these ducts were observed in adult animals (Xu et al., 2008; Li et al., 2010; Van de Casteele et al., 2013). Thus, Li and colleagues, when working on a model of partial PPx in Sprague-Dawley rats, found a focal decrease in the expression of one cut homeobox 1 (*Onecut1*, formerly known as Hnf6; a marker of ductal differentiation) in mature ductal cells, with a further transient appearance of regeneration areas in which new pancreatic lobes were generated later (Li et al., 2010). In these areas of regeneration, the researchers observed a transient expression of *Pdx*1, HNF1 homeobox B (*Hnf1b*, formerly known as *Tcf*2), *Sox*9, and then *Ngn*3. In addition, the forming islets were initially characterized as negative for *Mafa* expression (*Mafa* ^−^), but as they matured, the number of cells positive for both *Mafa* and *Ins* (*Mafa* ^+^/*Ins* ^+^) increased. According to Van de Casteele and colleagues, during murine pancreatic regeneration after PDL in *Ngn*3^CreERT^; Rosa26 locus^YFP^ mice, the number of pancreatic islets increased, and β-cell proliferation dominated. The β-cell proliferation potential was the highest in small islets, and up to 14% of all β-cells and 40% of small islet β-cells originated from non-β-cells (Van de Casteele et al., 2013).

In a recent study, Gribben and colleagues observed the presence of β-cell neogenesis from *Ngn*3-traced ductal cells in the adult pancreas of transgenic mice (Gribben et al., 2021). The authors showed that *Ngn*3^+^ cells can become *Ins* ^+^ cells through the intermediate state of somatostatin (*Sst*) positive cells. The authors found bihormonal (Ins^+^/Sst^+^) and monohormonal (Ins^+^ or Sst^+^) cells in the ducts, which sometimes aggregated into small islet-like clusters. A study using scRNA-seq has allowed us to hypothesize the ductal origin of some of the traced islet cells and predict that Ins+/Sst+ cells are intermediate for newly formed β-cells. This suggests that pancreatic ducts contain a population of progenitor cells that can differentiate into various endocrine cell types. 3D imaging revealed traceable *Ngn*3^+^ cells and *Ins* ^+^/*Sst* ^+^ cells located between ducts and islets, as would be expected of endocrine progenitor cells of ductal origin migrating to islets. Thus, this study suggests that β-cell neogenesis can occur in the ductal compartment at a rate of 0.66%–0.68%/week. Although this rate of β-cell neogenesis may seem low, over the course of a year, the cell mass increase of newly generated β-cells would be up to 30% (Gribben et al., 2021). In order to verify whether these cells are indeed a true source of new functional beta cells, it is necessary to observe their differentiation over a longer postnatal period (Gribben et al., 2021; Gribben et al., 2023). The results of this study were found to be ambiguous in the article by Magenheim and colleagues, who argue that Hnf1b-CreERT tags directly affect δ and β-cells in islets (Magenheim et al., 2023). The authors disagree with the conclusions of Gribben et al. that ductal Ngn3-expressing progenitors contribute to adult β-cell neogenesis in the pancreas and that neogenesis is enhanced in diabetes. Their own findings support the possibility that the *Neurog3* gene is active in pancreatic islet δ-cells and that the two Cre lines that were used directly label adult islet somatostatin-producing δ-cells, which precludes their use to assess whether β-cells originate from duct cells. Thus, Magenheim and colleagues stated that a weight of evidence indicates that islet cells are generated by the proliferation of differentiated cells and that neogenesis from ducts does not occur in the adult pancreas.

The hypothesis of possible pancreatic β-cell generation from endocrine progenitors in the pancreatic ducts has a long history (reviewed in Bonner-Weir et al., 2010; Granger and Kushner, 2009), and although there have always been studies not supporting the possibility of neogenesis. Though a recent study by Gribben has provided clear and convincing evidence for the possibility of neogenesis in mice (Gribben et al., 2021; Gribben et al., 2023), there have always been studies that reject the possibility of neogenesis in the adult pancreas (Magenheim et al., 2023). At the same time, in humans, the possibility of β-cell neogenesis and the origin of newly formed β-cells in the postnatal period also remain controversial and difficult to prove.

### 4.2 β-cell proliferation and cell cycle regulation

It is known that β-cell proliferation occurs early in embryonic development and continues through the neonatal stages. As β-cells mature, they enter a G0 quiescent state (Meier et al., 2010; Gregg et al., 2012). During embryogenesis and postnatally, the number of proliferating β-cells decreases from 3% of fetal cells to less than 0.5% (Rankin and Kushner, 2009; Perl et al., 2010). In the postnatal organism, β-cells have a low replication rate, which in mice and humans is 0.1%–0.2% (Teta et al., 2005; Meier et al., 2008; Gregg et al., 2012; Kulkarni et al., 2012; Wang et al., 2015). This low rate is, however, sufficient to maintain a stable β-cell mass under normal physiological conditions. A recent study has shown that many islet β-cells are the same age as the animal itself. Of note, there is no correlation between the age of β-cells and their cellular location within the islet, and rare instances of β-cell proliferation are random within the ultrastructure of the islet (Arrojo E Drigo et al., 2019). Thus, as shown in numerous studies, in the adult organism, β-cell replication is reduced to an almost undetectable level (Zhao et al., 2021). This fact may be explained by evolutionary mechanisms that prevent an unregulated increase in β-cell mass, which could cause hyperinsulinemia and lead to dangerous hypoglycemia, exemplified by endocrine carcinomas, insulinomas, or nesidioblastosis (Dardano et al., 2020).

Furthermore, it has been observed that β-cells in adults exhibit a reduced capacity for active proliferation and require a longer period of stimulation before they begin to divide. Thus, β-cells from 6-month-old ICR mice required 13 h of glucose stimulation to switch to proliferation, while β-cells from 5-week-old mice required only 8 h (Hija et al., 2014). Postnatal β-cells are characterized by increased expression levels of cell cycle inhibitors such as *Cdkn2a* and a decreased expression of cell cycle activators, including *FoxM1*, cyclins, and cyclin-dependent kinases (Avrahami et al., 2015; Golson et al., 2015; Tschen et al., 2017). At the same time, the population of proliferating β-cells shares similarities with immature β-cells in terms of gene expression (Nasteska et al., 2020; Shrestha et al., 2021). For example, when proliferation in the β-cells of adult transgenic mice was induced by exogenous expression of MYC proto-oncogene *(Myc*), these β-cells showed a reduced expression of genes important for GSIS *SLC*2*A*2, proprotein convertase subtilisin/kexin type 1 (*PCSK*1), as well as transcriptional markers of mature β-cells: *Pdx*1, *MafA*, and *Nkx*2.2 (Puri et al., 2018).

Mitogenic stimuli have been shown to “awaken” quiescent β-cells by controlling the expression and activation of specific cyclins and cyclin-dependent kinases (CDKs), allowing β-cells to re-enter the cell cycle (Martínez-Alonso and Malumbres, 2020). Mitogens that regulate the cell cycle and β-cell proliferation include a wide range of different factors such as insulin, nutrients (e.g., glucose, amino acids), growth factors (insulin-like growth factor I (IGF-1), hepatocyte growth factor (HGF), platelet-derived growth factor (PDGF), placental growth factor (PIGF)), incretins - glucagon-like peptide-1 (GLP-1), exendin-4 (Ex-4), liraglutide, glucose-dependent insulinotropic polypeptide (GIP)), leptin, estrogen, progesterone, prolactin and placental lactogen, and serpentine receptors (G protein-coupled receptors (GPCRs)) as reviewed in detail by Shcheglova and co-authors (Shcheglova et al., 2022). Signals from these inducers, e.g., Akt, mTOR, MAP kinases, and cAMP-PKA, in turn influence transcription regulators such as forkhead box O1 (*FoxO*1), STAT proteins, CREB, and β-catenin, as detailed in relevant reviews (Bernal-Mizrachi et al., 2014; Stewart et al., 2015).

Importantly, glucose, a key humoral factor for β-cells, itself has a mitogenic effect on β-cells (Porat et al., 2011). Stimulation of β-cells by glucose can lead to an increase in insulin secretion and a decrease in blood glucose levels. The rate of β-cell duplication is controlled by glycolysis and subsequent depolarization of the cell membrane due to the closure of ATP-sensitive potassium channels (Porat et al., 2011). In mice, a short-term, 4-day effect of moderate hyperglycemia resulted in an increase in β-cell replication. This increase was mediated, at least in part, by an increase in cyclin D2 (Alonso et al., 2007). Under the conditions of low glucose and low ATP, cell growth is inhibited, mainly through increased AMP-activated protein kinase (AMPK) activity (Yuan et al., 2013). However, at normal or high glucose concentrations, numerous signaling molecules important for cell growth are activated, including the mammalian target of rapamycin complex 1 (mTORC1) and protein kinase A (PKA) (Costes et al., 2004; Laplante and Sabatini, 2013; Linnemann et al., 2014).

The incretin hormones GLP-1 and GIP are peptide hormones produced by enteroendocrine cells in the intestine. The incretin hormones are known to transmit signals through the same signaling pathways as glucose and insulin. With regard to the incretins, especially GLP-1, which is a promising therapeutic agent, their role in inducing the proliferation and preventing the apoptosis of β-cells during obesity has been established (Fusco et al., 2017), both through the mechanism of stimulation of insulin secretion and through direct stimulation of β-cell replication, which can be enhanced thanks to a joint effect of incretin hormones and dual specificity tyrosine phosphorylation regulated kinase 1A (DYRK1A) inhibitors (Ackeifi et al., 2020). It has also been reported that GLP-1 is capable of inducing transdifferentiation of α-cells into pancreatic β-cells (Lee et al., 2018).

Glucagon-like peptide one receptor (GLP1R) ligands may act as mitogens to promote β-cell proliferation (Chen S. et al., 2014; Fusco et al., 2017). Exendin-4, an agonist of GLP1R, stimulates human β-cell proliferation in juvenile but not adult pancreatic islets, as shown in an elegant experiment by Dai and colleagues with transplantation of human islets under the mouse renal capsule (Dai et al., 2017). The mitogenic effect of Ex-4 involves a signaling pathway with calcineurin and nuclear factor of active T cells (NFAT) and induces the expression of target genes for proliferation-promoting factors including nuclear factor of activated T cells 1 (*NFATC*1), *FOXM*1, Cyclin A1, and *CDK*1 in juvenile islets. Interestingly, Ex-4 stimulates insulin secretion by both juvenile and postnatal human β-cells, i.e., sensitivity to Ex-4 persists with age, but the ability for intense proliferation disappears (Dai et al., 2017). Transcription factors Srf, Jun, and Fos identified by transcriptome analysis may also play a role in stimulating postnatal proliferation and increasing β-cell mass (Zeng et al., 2017).

Interestingly, many of the mitogens effective for stimulating human β-cells, such as garmin and its analogs, promote proliferation through inhibition of DYRK1A (Wang et al., 2015). DYRK1A phosphorylates NFAT, rendering it inactive and keeping it in an inactive state in the cytoplasm. As a result of DYRK1A inactivation, NFATs activate the expression of genes that promote cell cycle initiation: FOXM1, cyclin E1, cyclin A2, and CDK1, and suppress the expression of inhibitors such as p57, p15, and p16 (Wang et al., 2019). In a recent study, Wang and colleagues demonstrated that small-molecule inhibitors of DYRK1A induce human β-cells to proliferate (Wang et al., 2022). This group studied transcriptomic databases of insulinoma and human β-cells to understand why β-cells in insulinomas proliferate intensely. They identified the DREAM protein complex as a central regulator of the quiescence phase (G0, regulator of quiescence) in human β-cells. The DREAM complex consists of a module of transcriptionally repressive proteins that assemble in response to DYRK1A kinase activity, thereby inducing and maintaining the cellular quiescence phase. In the absence of DYRK1A, DREAM subunits reassemble into the pro-proliferative MMB complex (Wang et al., 2022).

An interesting area of research is constituted by different combinations of mitogens and inhibitors to increase their effect and, consequently, β-cell proliferation. For instance, combined pharmacological inhibition of DYRK1A and TGFβ results in a significant further synergistic 5%–8% boost in human β-cell proliferation and raises the number of β-cells in both mice and humans (Wang et al., 2019). However, despite the promise of mitogens and especially their combinations, there are a number of challenges in selecting mitogens that are effective and specific for human β-cells. Many effective mitogens that have been identified in rodents do not necessarily have similar efficacy when applied to human β-cells. Besides, most mitogens for β-cells also induce proliferation of α-cells and other endocrine cells (Shcheglova et al., 2022).

In most cases of T1DM, a small β-cell population is preserved (Campbell-Thompson et al., 2016). Consequently, it is also possible to stimulate the proliferation of these cells. Extensive studies have led to the identification of a number of human β-cell mitogens, but their efficiency and specificity remain insufficient compared with their effect on rodent cells. The strategy of using a combination of several mitogens looks promising. Current data on this topic have been collected and systematized in comprehensive reviews (Angueira et al., 2015; Sferruzzi-Perri et al., 2020; Shcheglova et al., 2022). At the same time, forced stimulation of a cell to mitosis may induce tumorigenesis, since the action of mitogens has similarities with the disruption of intrinsic anti-oncogenic mechanisms. To minimize these risks, proliferation should be highly specific for β-cells, short-term, and strictly controlled.

### 4.3 Dedifferentiation of β-cells in pathological conditions and the possibility of subsequent redifferentiation into functional β-cells

Diabetes of various etiologies is characterized by a decrease in insulin expression. This decrease may result from β-cell damage or dedifferentiation. Dedifferentiation may confer resistance to stress factors, such as an autoimmune response or lipotoxicity (Cinti et al., 2016; Bensellam et al., 2018). In some cases, there is marked dedifferentiation of β-cells to a state similar to progenitor cells, with a loss of phenotype and cellular identity of β-cells (Amo-Shiinoki et al., 2021; Khin et al., 2021; Nimkulrat et al., 2021; Francis et al., 2023). Occasionally, cells with low levels of insulin expression remain, but they are not dedifferentiated β-cells (Lam et al., 2019). An important consideration is whether a population of dedifferentiated β-cells can maintain the ability to redifferentiate into a fully functional active state with continued exposure to a stress agent. T2DM is reversible in the early stages of the disease; nevertheless, the ability to recover a functional cell mass is known to decrease with increasing disease duration (Hallberg et al., 2019). Importantly, in the absence of expression of four major islet hormones, the number of cells that remain immunoreactive to the pancreatic islet endocrine cell marker chromogranin A (*CHGA*) increases in diabetic islets and increases significantly during disease progression (Amo-Shiinoki et al., 2021). Moreover, the loss of the ability of β-cells to synthesize insulin and their dedifferentiation to a cell type resembling endocrine progenitors is induced by a decreased activity of the transcription protein FoxO1. It is possible that these dedifferentiated cells are capable of developing into functional β-cells when metabolic stress factors are removed (Casteels et al., 2021). Although the major role of glucolipotoxicity in β-cell dedifferentiation is well established, the exact mechanisms involved in this process are still under investigation, as reviewed in detail by Lytrivi and colleagues (Lytrivi et al., 2020).

Thus, in various pathological conditions, the reduction in the mass of functionally active β-cells is caused not only by cell death but also by the dedifferentiation of β-cells. The loss of the mature β-cell phenotype is closely linked to the loss of insulin secretion ability. This, in turn, leads to the progression of the pathological condition. Pharmacological and cell-based therapies (e.g., allogeneic *postmortem* islet transplantation [Mullard, 2023]) combat the consequence in the form of insulin deficiency rather than the primary cause, i.e., the loss of native functional β-cells. The possibility of redifferentiation of dysfunctional islets is one of the most promising approaches to diabetes therapy.

### 4.4 Transdifferentiation from other cell types into pancreatic β-cells

Transdifferentiation is defined as the transition from one cell type to another without passing through the intermediate stage of pluripotent or progenitor cells. Pancreatic endocrine cells are known to represent a stable, terminally differentiated cell population. However, research has shown that under conditions of stress or genetic manipulation, they exhibit a high degree of plasticity. There is wide-ranging evidence of the transition of different cell types into β-like cells (Papizan et al., 2011; Ediger et al., 2017; Gutiérrez et al., 2017; Swisa et al., 2017). On the other hand, one study has shown that non-insulin-secreting cells, of both endocrine and exocrine origin, do not generate new β-cells in the postnatal pancreas, whether in normal health or at and after PDL and PPx and ablation of pancreatic β-cells with streptozotocin. However, within the exocrine compartment of the pancreas, the appearance of *Ins* ^+^ cells from non-β-cells was observed after genetic ablation of 99% of β-cells, consistent with a transdifferentiation scenario (Zhao et al., 2021).

Early studies on transdifferentiation detected the emergence of β-like cells from other islet cell types and acinar cells, often involving bihormonal cells, which are likely to be intermediate cells that later acquire a β-like phenotype (Figure 1B). Thus, bihormonal β-like cells co-expressing somatostatin and insulin were described in pancreatic islets after STZ-induced β-cell death. These β-like cells co-express somatostatin and insulin (Fernandes et al., 1997). Cells co-expressing the genes of different hormones have also been found in the embryonic development of the murine and human pancreas (Riedel et al., 2012; Perez-Frances et al., 2022). Studies on transgenic mouse models have demonstrated that only adult β-cells are formed from embryonic bihormonal for insulin, glucagon (Gcg), somatostatin, or pancreatic polypeptide cells (Ins^+^/Gcg^+^, Ins^+^/Sst^+^, and Ins^+^/Ppy^+^) during development. The expression of insulin is indicative of terminal differentiation into β-cells (Perez-Frances et al., 2022). Aristaless-related homeobox (*ARX*) positive cells are also known to co-produce insulin and glucagon in the absence of expression of transcription factors *PDX1*, *NKX*6.1, and *MAFA* (Riedel et al., 2012). Cells bihormonal for insulin and glucagon have similarly been found in people with T1DM and T2DM (Md Moin et al., 2016; Chakravarthy et al., 2017).

A potential cellular source for β-cell regeneration within the pancreas itself, which has been the focus of much research in the field of transdifferentiation, is a population of endocrine α-cells (Figure 1B). On the other hand, some authors did not find a direct transdifferentiation from the α-cell to the β-cell lineage in the STZ model of diabetes (Feng et al., 2020). In a transgenic mouse model, it was shown that upon a near total ablation of β-cells, α-cells began to co-express insulin. Some of these bihormonal cells eventually become monohormonal Ins^+^ cells (Thorel et al., 2010). The main transcription factor involved in maintaining the α-cell identity is Arx. However, there are a number of other genes involved in determining α-cell identity, such as regulator of glucagon expression brain 4 (Brn4) (Hussain et al., 1997) or DNA methyltransferase 1 (Dnmt1) as well as maintenance of other epigenetic marks. Genetic lineage tracing and scRNA-seq revealed that the loss of *Dnmt*1 and *Arx* results in the transformation of α-cells into a population of β-like cells. These cells resemble β-cells in their transcriptomic profile but have a lower insulin secretion capacity compared to native β-cells (Chakravarthy et al., 2017). Interestingly, *Arx* inhibition results in a continuous mobilization of duct lining progenitor cells, which acquire features of *Ngn*3^+^ endocrine progenitors (Courtney et al., 2013). In a recent study, RNA-seq of α-cells following β-cell ablation in mice showed that α-cells undergo specific changes in their transcriptional programs (Oropeza et al., 2021). The authors confirmed the temporal modulation of Smoothened and Insulin signaling pathways, which can be possibly involved in transdifferentiation towards insulin-producing cells (Oropeza et al., 2021).

There is another approach to utilizing α-cells as a cell source for transdifferentiation into β-like cells, based on the regulation of the activity of genes directly related to various β-cell transcription factors. For example, paired box 4 (PAX4) gene is a regulator of β-cell factors such as *PDX*1, *MAFA*, *NGN*3, and *NKX*6.1 (Zhang et al., 2016). *PAX*4 ectopic expression triggers a cycle of neogenesis and transdifferentiation of α-cells into β-like cells that leads to glucagon deficiency. This promotes compensatory and continuous neogenesis of *INS* ^+^ cells, which cannot correct hypoglucagonemia as they subsequently acquire the phenotype of β-cells. As a result, there is a significant level of restitution of functional β-cell mass capable of correcting chemically induced diabetes in animals (Collombat et al., 2009). In a 2023 study performed on zebrafish, the authors were able to show the presence of transdifferentiation of ghrelin-expressing ε-cells into β-cells both during embryogenesis and after an almost complete ablation of β-cells. Transdifferentiation of ε-cells into β-cells was enhanced by deletion of *Pax*4, which suppresses ghrelin expression and leads to the formation of more ghrelin^+^ cells, consequently potentiating β-cells regeneration (Yu et al., 2023). Another transcription factor, *PAX*6, is a transcription factor that activates β-cell genes, maintaining the functional activity and identity of mature β-cells. Deletion of *Pax6* in adult murine β-cells leads to lethal hyperglycemia and ketosis, probably due to a loss of β-cell function and to an expansion of α-cell (Swisa et al., 2017).

Other factors whose ectopic expression can lead to the transformation of cells into β-like cells are the insulin gene regulators *PDX*1 and *MAFA* (Zhu et al., 2017). Thus, transduction of human α-cells with an adenovirus expressing *PDX*1 and *MAFA* resulted in the transdifferentiation of approximately 35% of these α-cells into *INS* ^+^ cells. Moreover, transplantation of pseudo-islets derived from these cells into diabetic immunodeficient NSG RIP-DTR mice after diphtheria toxin administration resulted in improved insulin secretion and reduced glucose tolerance (Furuyama et al., 2019). Interestingly, α- and γ-cells derived from post-mortem human donor islets from both healthy and diabetic donors can also be reprogrammed by transcription factors *PDX*1 and *MAFA* into insulin-secreting cells (Furuyama et al., 2019). Furthermore, induced expression of *PDX*1 and *MAFA* leads to the development of a β-like cell phenotype in both α-cells and *Ngn*3^+^ endocrine progenitor cells (Matsuoka et al., 2017). In fetal and adult transgenic mice, specific deletion of *FoxO*1 in *Ngn*3^+^ enteroendocrine progenitors resulted in increased expression of transcription factors *Pdx*1, *Ngn*3, *MafA*, and *Nkx*6.1, turning the progenitor cells into *Ins* ^+^ cells (Talchai et al., 2012).

The accumulating knowledge about the mechanisms of transdifferentiation of different cell types also allows us to think about the possibility of combining strategies, cellular sources, and transcription factors to achieve the most efficient way of obtaining insulin-secreting cells. It is known that pancreatic acinar cells can transdifferentiate into different types of pancreatic islet cells, which can then give rise to a population of insulin-secreting cells. Expression of *Ngn*3 in murine acinar cells induced their transdifferentiation into δ-cells, and co-expression of *Ngn*3 and *MafA* led to the transformation of acinar cells into α-like cells (Li et al., 2014), thus highlighting the perspective of transdifferentiation of acinar cells into different types of endocrine cells as well. It is worth noticing the importance of considering the role of a huge number of factors that influence the final outcome of transdifferentiation. For example, hyperglycemia suppresses the reprogramming of acinar cells into β-cells by a combination of transcription factors *Pdx*1, *Ngn*3, and *MafA* (PNM factors), highlighting the importance of glucose levels for the regenerative potential of β-cells and the plasticity of the exocrine pancreas (Cavelti-Weder et al., 2016).

Some very important work was carried out by Zhou and colleagues in 2008. The authors screened various combinations of critical transcription factors whose expression is observed in β-cells or their progenitors *in vivo*. By analyzing combinations of factors, it was determined that a combination of three PNM factors is sufficient for the transdifferentiation of pancreatic exocrine cells into insulin-producing β-like cells (Zhou et al., 2008). The PNM combination was able to transform acinar cells into β-like cells capable of reducing hyperglycemia in mice with toxin-induced diabetes (Clayton et al., 2016). In immunodeficient (NOD-SCID) mice, adenoviral vector-mediated delivery of PNM also induced differentiation of *SOX9* ^+^ ductal cells in the liver toward insulin-producing cells (Banga et al., 2014). In addition, forced expression of *PDX*1, *NGN*3, and *MAFA* in murine intestinal crypt cells and human intestinal organoids converted them into β-like cells. Furthermore, in this study, expression of *PDX*1, *NGN*3, and *MAFA* in intestinal cells also resulted in a moderate but significant improvement in glucose tolerance levels in streptozotocin-treated transgenic mice (Chen Y.-J. et al., 2014).

Although reprogramming cells to induce insulin secretion is an attractive possibility for diabetes therapy, the molecular mechanisms underlying the process of transdifferentiation of endocrine cells of the pancreatic islets into β-cells in humans have not been fully elucidated. The transdifferentiation of different cell types into β-cells for the treatment of diabetes mellitus is still a long way off.

## 5 The pancreas during pregnancy and obesity

The functional mass of pancreatic β-cells increases in response to pregnancy and obesity. It is well-established that β-cell mass can increase by 20%–90% in obese individuals without diabetes (Linnemann et al., 2014; Inaishi and Saisho, 2020). The second specific condition under which pancreatic β-cell proliferation can be observed is undoubtedly maternal adaptation to pregnancy. The pancreas’ ability to self-regulate in response to increasing insulin demand is reflected in β-cell proliferation and increased β-cell mass during pregnancy and obesity. This phenomenon is of great interest for the identification of mediators determining proliferation, survival, and the maintenance of functional β-cell mass.

### 5.1 β-cell mass increases in obesity

Obesity contributes significantly to the increase in β-cell mass by enhancing β-cell proliferation (Linnemann et al., 2014; Inaishi and Saisho, 2020). In rodents, a significant increase in β-cell mass is also observed in obesity caused by diet or mutations in certain genes (Hull et al., 2005). For example, a threefold increase in β-cell mass was observed in young mice with obesity induced by a high-fat diet (HFD), but not in older mice. The authors of this study (Tschen et al., 2009) also found a correlation between the level of BMI1 proto-oncogene (*Bmi1*) gene expression and the ability of the β-cell mass to increase, as well as a reduced level of *Cdkn*2*a* expression in pancreatic β-cells.

Adipose tissue secretes a wide range of hormones, mediators, and growth factors called adipokines that can directly influence β-cell function, viability, and proliferation (Biondi et al., 2022). For example, leptin levels are a precise regulator of β-cell mass through participation in many signaling pathways (Frühbeck, 2006). At normal concentrations, leptin reduces fatty acid-induced apoptosis of β-cells and stimulates β-cell proliferation *in vitro*, probably through activation of mitogen-activated protein kinase (MAPK) (Morioka et al., 2007). However, high levels of leptin and glucose induce β-cell apoptosis via the JNK pathway. Prolonged exposure of human islets to leptin also leads to impaired β-cell function, caspase-3 activation, and increased levels of apoptosis (Biondi et al., 2022). Such findings are supported by experiments on mice with leptin gene knockout, leading to impaired GSIS but increased β-cell mass (Covey et al., 2006).

Other adipokines affecting β-cells include irisin, visfatin, apelin, and resistin (Biondi et al., 2022). Irisin, a hormone secreted by skeletal muscle, promotes β-cell proliferation, most probably through activation of extracellular signal-regulated kinase (ERK1/2) and p38 MAPK, and increases β-cell mass and proliferation (Liu et al., 2017; Marrano et al., 2021; 2022). Visfatin (Cheng et al., 2011; Kim et al., 2014) and apelin (Feng et al., 2019) may also be involved in the induction of β-cell proliferation *in vitro* and in rats in HFD and STZ models of diabetes. Apelin can maintain β-cell identity in mice in the HFD and STZ models of diabetes, ensuring maintenance of β-cell mass, and also contributes to a decrease in apoptosis and to increased β-cell proliferation (Gao et al., 2018; Tanday et al., 2020). Resistin may also be a factor inducing β-cell proliferation (Park et al., 2008) increasing β-cell viability by decreasing insulin receptor expression levels (Brown et al., 2007). However, mice with increased resistin expression have an impaired insulin secretory response to glucose (Nakata et al., 2007).

The phenomenon of increasing β-cell mass during obesity is achieved by a complex of factors. The increase in β-cell volume in obesity is mainly due to the replication of existing β-cells within islets rather than the formation of new islets. It is unlikely that neogenesis or transdifferentiation of other cell types into β-cells contributes to the compensatory increase in β-cell mass.

### 5.2 The cellular mechanisms of β-cell adaptation to pregnancy

During pregnancy, the maternal pancreas undergoes adaptation, resulting in the proliferation of pancreatic beta cells. The mass of beta cells increases, and their functional activity is elevated to meet the metabolic needs of both the mother and the growing fetus (Van Assche et al., 1978; Rieck et al., 2009; Butler et al., 2010; Sylvester-Armstrong et al., 2023). A key aspect of maternal metabolic adaptations is a decrease in peripheral tissue sensitivity to insulin (Zeng et al., 2017; Taschetto et al., 2021). Substances secreted by the placenta affect insulin tolerance and pancreatic adaptation. For example, progesterone can increase lipolysis and decrease insulin sensitivity and glucose uptake in peripheral tissues. Additionally, human placental growth hormone is involved in β-cell adaptation to the body’s metabolic needs. Prolactin (PRL) and placental lactogen (PL) also influence pregnancy-related β-cell adaptation to peripheral insulin resistance and increased lipolysis (Moyce and Dolinsky, 2018; Moyce Gruber and Dolinsky, 2023).

Studying β-cell adaptation during human pregnancy is challenging due to the inaccessibility of pancreatic samples. Therefore, many studies have been conducted on rodents. In mice, the peak of proliferation occurs between days E11 and E15. On day E14.5, there is a 3–4-fold increase in β-cell mass and a 3-fold increase in β-cell size (Rieck et al., 2009). An active generation of new β-cells in murine islets during pregnancy was evidenced by the high number of β-cells including 5-Ethynyl-2′-deoxyuridine (EdU) during pregnancy: 3.7% in pregnancy versus 0.4% in controls (Zhao et al., 2021).

In Wistar rats, there was an increase in pancreatic β- and α-cell mass by the end of the third week (Gallego et al., 2018). The few existing studies on humans have shown that β-cell mass increases approximately 1.4–2.4-fold in pregnant women, which is less than in mice (Van Assche et al., 1978; Butler et al., 2010; Sylvester-Armstrong et al., 2023). During pregnancy in humans, the number of pancreatic islets and β-cells in them increases, and the average size of the islets decreases. It is likely that the appearance of new medium-size islets contributes significantly to the increase in β-cell mass during pregnancy in humans, as opposed to rodents.

A recent study by Sylvester-Armstrong and colleagues showed the presence of β-cell proliferation *in vitro*, when cells were cultured in a medium containing the blood serum of pregnant women (Sylvester-Armstrong et al., 2023). The statement suggests that there are factors related to pregnancy that induce the proliferation or neogenesis of pancreatic β-cells. The factors involved in this process include endocrine factors such as PL, PRL, and growth hormone, as well as growth factors such as HGF, PIGF, and EGF. The entire set of known molecular mechanisms of β-cell proliferation is extensively reviewed by Salazar-Petres (Salazar-Petres and Sferruzzi-Perri, 2022). Elevated levels of placental lactogens and prolactin are important factors in increasing islet cell mass and β-cell proliferation. These factors trigger β-cell proliferation through the prolactin receptor (PRLR), which activates the JAK-STAT pathway (Salazar-Petres and Sferruzzi-Perri, 2022). PRL induces expression of PDZ-binding kinase Pbk through activation of the JAK-STAT pathway, and STAT5 binds to the Pbk locus to activate its transcription in INS^+^ islet cells in pregnant mice on E15.5. Deactivation of Pbk kinase reduces adaptive proliferation and β-cell mass (Cao et al., 2021). Moreover, pregnant mice lacking PRLR in β-cells develop gestational diabetes, and STZ diabetic mice lacking PRLR showed increased intensity and frequency of hyperglycemia, decreased pancreatic islet density, β-cell proliferation, survival, and circulating insulin levels (Ramirez-Hernandez et al., 2021). Analysis of the transcriptome of murine pancreatic islets during pregnancy revealed a key role of PRL in activating a cascade of genes responsible for cell proliferation and β-cell survival (Pepin et al., 2019). The dramatic increase in rodent β-cell proliferation and mass during pregnancy is also associated with the activation of signaling pathways such as Raf/MEK/ERK and PI3K/AKT, various transcription factors including *FoxM*1, *Hnf*4*a*, forkhead box D3 (*Foxd*3), BCL6 transcription repressor (*Bcl*6), and *Mafb*, epigenetic regulators such as menin and enhancer of zeste two polycomb repressive complex two subunit (*Ezh*2), serotonin metabolic pathways, and changes in calcium dynamics in various subcellular compartments of β-cells (Pretorius and Huang, 2022).

It is hypothesized that one of the cellular sources of new β-cells is constituted by specific populations of postnatal pancreatic progenitors. Zhao and colleagues suggest that no β-cell neogenesis occurs from progenitors or stem cells during pregnancy or after PDL, PPx and ablation of pancreatic β-cells with streptozotocin (Zhao et al., 2021).

Data on β-cell populations during pregnancy are also extremely limited; Dirice and colleagues in 2019 identified ductal cells positive for markers of immature β-cells (*SOX*9, *NEUROD*1, *PAX*6, *PDX*1, and *MAFA*) in pancreatic sections from pregnant women and in people with T2DM (Dirice et al., 2019). They also found an increase in the number of *Ins* ^+^/*Gcg* ^+^ ductal cells along with the presence of small clusters of islets composed of *Ins* ^+^ and *Gcg* ^+^ cells immediately adjacent to or very close to ductal cells throughout the pancreas in pregnant mice. Thus, under increased insulin demand, ductal cells contribute to the compensatory pool of β-cells through differentiation or neogenesis. In contrast, Liu’s research group found that some of the pancreatic ductal cells became *Ins* ^+^ on E16 in mice. However, these *Ins* ^+^ cells were not fully functionally mature β-cells, as they did not respond to glucose *in vitro* and showed low expression levels of key β-cell genes. These Ins^+^ cells found in the ducts have exhibited lower expression levels of genes linked to extracellular matrix degradation and cell migration. This may prevent them from “budding” and migrating into established islets. Liu and colleagues suggest that *Ins* ^+^ ductal cells make little or no contribution to the population of functional β-cells (Liu et al., 2021). Strutt and colleagues found that the number of bihormonal Ins^+^/Glut^low^ β-cells increases three-to fourfold during pregnancy, reaching a maximum on days 9–12 of gestation in C57B6/6J mice (Strutt et al., 2021). As described above, Bader and colleagues identified *CFAP*126^−^ β-cells. During pregnancy, they are able to proliferate and then change their expression profile to *CFAP*126^+^ for better management of metabolic changes. *CFAP*126^−^ β-cells have a greater proliferative potential and are able to further change their expression profile to *CFAP*126^+^, characterized by increased GSIS, to more efficiently manage metabolic changes (Bader et al., 2016).

Summarizing, to balance maternal processes during pregnancy, compensate for insulin resistance, and avoid excessive hyperglycemia and glucose intolerance, maternal pancreatic β-cells undergo significant alterations in their functions, such as GSIS and regulation of β-cell proliferation and death rates (Rieck and Kaestner, 2010). Active generation of new β-cells during pregnancy was evidenced in both rodents and humans. Important factors increasing islet cell mass and β-cell proliferation include prolactin, placental lactogens, and growth hormone, as well as a number of growth factors. The studies focusing on progenitor cells and the generation of Ins^+^ cells from them demonstrate contradictory results and are extremely limited, as well as data on β-cell populations during pregnancy.

## 6 Conclusion

The phenomenon of β-cell heterogeneity in terms of transcription profiles and hormone secretion regulation is extremely important in the context of identifying cellular sources and inducers of functional β-cell renewal. Under stress conditions, certain β-cell populations have the capacity to survive by losing the phenotype of mature β-cells. Dedifferentiated β-cells contribute to a decrease in the total number of functionally active β-cells but are also a potential source of self-reproduction due to their ability to redifferentiate into a functionally active state. Transdifferentiation of different cell types into insulin-secreting cells is another attractive possibility for developing approaches to diabetes therapy. However, the cellular and molecular mechanisms of these phenomena are not completely clear.

The increased functional mass of pancreatic β-cells in pregnancy and obesity is of great interest. The proliferation of β-cells observed during pregnancy is the result of maternal adaptation and reflects the pancreas’s ability to self-regulate in response to increased insulin demand. Similarly, in obesity, an increase in β-cell mass satisfies the elevated insulin demand. High levels of placental lactogens and prolactin during pregnancy, as well as the adipokine complex in obesity without diabetes, are important factors that affect increased islet cell mass and β-cell proliferation. Nevertheless, the identification of markers of mature β-cell populations and postnatal pancreatic progenitors capable of intensifying replication in obesity and pregnancy remains a challenge.

In summary, the existence of various scenarios and mechanisms for β-cell recovery under different conditions (physiological, pathological, and in experimental animal models) suggests a significant diversity of β-cell populations. Currently, there are limited practical studies that link the heterogeneity of β-cell populations and their diversity of reactions to factors inducing regeneration into a single theme with the issue of β-cell regeneration under different physiological conditions. All this makes it even more important to analyze such studies, which in the future may give rise to the development of specific approaches for the correction of various pathologies associated with pancreatic β-cells.

## Figures and Tables

**Table:** 

**Abcc8**	ATP-binding cassette subfamily C member 8
**AMPK**	AMP-activated protein kinase
**ARX**	aristaless-related homeobox
**Bcl6**	BCL6 transcription repressor
**Bmi1**	BMI1 proto-oncogene
**BrdU**	bromodeoxyuridine
**Brn4**	brain 4
**Cdkn2a**	cyclin dependent kinase inhibitor 2A
**CDKs**	cyclin-dependent kinases
**Cfap126**	cilia and flagella associated protein 126
**CHGA**	chromogranin A
**CHGB**	chromogranin B
**DDIT3**	DNA damage inducible transcript 3
**Dnmt1**	DNA methyltransferase 1
**DYRK1A**	dual specificity tyrosine phosphorylation regulated kinase 1A
**EdU**	5-Ethynyl-2′-deoxyuridine
**ENTPD3**	ectonucleoside triphosphate diphosphohydrolase 3
**ER**	endoplasmic reticulum
**ERK1/2**	extracellular signal-regulated kinase
**ERO1LB**	endoplasmic reticulum oxidoreductase 1 beta
**Ex-4**	Exendin-4
**Ezh2**	enhancer of zeste 2 polycomb repressive complex 2 subunit
**fos**	Fos proto-oncogene
**FOXA2**	forkhead box A2
**Foxd3**	forkhead box D3
**FoxM1**	forkhead box M1
**FoxO1**	forkhead box O1
**G6PC2**	glucose-6-phosphatase catalytic subunit 2
**Gcg**	glucagon
**GIP**	glucose-dependent insulinotropic polypeptide
**GLP-1**	glucagon-like peptide-1
**GLP1R**	glucagon-like peptide 1 receptor
**GPCRs**	G protein-coupled receptors
**GSIS**	glucose-sensitive insulin secretion
**HDAC9**	histone deacetylase 9
**HERPUD1**	homocysteine inducible ER protein with ubiquitin like domain 1
**HFD**	high-fat diet
**HGF**	hepatocyte growth factor
**Hnf1b**	HNF1 homeobox B
**HSPA5**	heat shock protein family A (Hsp70) member 5
**IAPP**	islet amyloid polypeptide
**IGF-1**	insulin-like growth factor I
**INS**	human insulin gene
**Ins2**	mouse insulin gene
**ISL1**	ISL LIM homeobox 1
**Jun**	Jun proto-oncogene
**KLF9**	KLF transcription factor 9
**Mafa**	MAF bZIP transcription factor A
**MafB**	MAF bZIP transcription factor b
**MAPK**	mitogen-activated protein kinase
**MODY**	maturity onset diabetes of the young
**mTORC1**	mammalian target of rapamycin complex 1
**Myc**	MYC proto-oncogene
**Neurod1**	neuronal differentiation 1
**Neurog3**	neurogenin-3
**NFAT**	nuclear factor of active T cells
**NFATC1**	nuclear factor of activated T cells
**NKX2.2**	NK2 homeobox 2
**Nkx6.1**	NK6 Homeobox 1
**NOD mice**	non-obese diabetic mice
**NPY**	neuropeptide Y
**Onecut1**	one cut homeobox 1
**PAX4**	paired box 4
**PCSK1**	proprotein convertase subtilisin/kexin type 1
**PDGF**	platelet-derived growth factor
**PDL**	pancreatic duct ligation
**Pdx1**	pancreatic and duodenal homeobox 1
**PIGF**	placental growth factor
**PKA**	protein kinase A
**PL**	placental lactogen
**PNM factors**	Pdx1, Ngn3, and MafA
**PP**	pancreatic polypeptide
**PPx**	partial pancreatectomy
**PRL**	Prolactin
**PRLR**	prolactin receptor
**PSCs**	pluripotent stem cells
**Ptf1a**	pancreas Associated Transcription Factor 1a
**scRNA-seq**	scRNA-sequencing
**SHISAL2B**	shisa like 2B
**SLC2A2**	solute carrier family 2 member 2
**Sox9**	SRY-Box Transcription Factor 9
**Srf**	serum response factor
**Sst**	somatostatin
**ST8SIA1**	ST8 alpha-N-acetyl-neuraminide alpha-2,8-sialyltransferase 1
**STZ**	streptozotocin
**T1DM**	type I diabetes mellitus
**T2DM**	type II diabetes mellitus
**Ucn3**	urocortin 3
**UPR**	unfolded protein response
**ZNT8**	solute carrier family 30 member 10
